# TNF-α blockade is ineffective in animal models of established polycystic kidney disease

**DOI:** 10.1186/1471-2369-14-233

**Published:** 2013-10-25

**Authors:** Jeffrey Roix, Saurabh Saha

**Affiliations:** 1 Biomed Valley Discoveries Inc, 4520 Main Street, Suite 1650, MO 64111, Kansas City, USA

**Keywords:** Polycystic kidney disease, Etanercept, TNF-α, PKHD1, PKD2

## Abstract

**Background:**

Given the large medical burden of polycystic kidney disease (PKD) and recent clinical trial failures, there is a need for novel, safe and effective treatments for the disorder.

**Methods:**

In PCK rat and PKD2^(ws25/w183)^ mouse models, entanercept was administered once every three days at 5 or 10 mg/kg, once daily. Mozavaptan was administered as a pilot control, provided continuously via milled chow at 0.1%. Animals were assessed for measures of pharmacodynamic response, and improvements in measures of polycystic kidney disease.

**Results:**

Entanercept treatment modulated inflammatory markers, but provided limited therapeutic benefit in multiple animal models of established polycystic kidney disease. Kidney weight, cyst burden and renal function markers remained unchanged following administration of etanercept at various dose levels and multiple treatment durations.

**Conclusions:**

While it remains possible that TNF-α inhibition may be effective in truly preventative settings, our observations suggest this pathway is less likely to exhibit therapeutic or disease-modifying efficacy following the standard clinical diagnosis of disease.

## Background

As many as 1 in 20 patients on dialysis suffer from polycystic kidney disease: this chronic, progressive disorder emerges over years in patients who carry a spectrum of well-characterized genetic alterations [1]. Autosomal dominant and recessive mutations in the polycystin gene family (PKHD1, PKD2) are thought to cause an array of defects in basic renal epithelial cell function, which in turn culminate in the hallmark cysts and kidney dysfunctions characteristic of the disease [2,3].

Despite an understanding of both pathophysiologic and correlative molecular genetic processes, effective and safe treatments for PKD are not currently available, and recent interventional trials for the disease have been disappointing. Preclinical studies highlighted the possible value of targeting the vasopressin and mTOR/AKT pathways [4-6]. Several PKD clinical trials assessing drugs that target these pathways, however, have suggested only marginal efficacy, in the face of appreciable side effects [7-9].

In this setting, it is promising that studies of basic biological processes associated with PKD pathophysiology have yielded new therapeutic targets. TNF-α is a canonical immune cytokine that activates inflammatory signaling in disorders ranging from arthritis to bowel disease [10,11]. Recently, this pathway was implicated in fundamental cellular processes of PKD: active TNF-α interferes with processing and membrane presentation of PKD2, effectively phenocopying genetic ablation of the disease locus. TNF-α was shown to be a both necessary and sufficient modifier of polycystic kidney disease. Intriguingly, TNF-α inhibition, using the approved biologic drug etanercept, could prevent kidney cyst formation in animals heterozygous for PKD2 deficiency that are normally predisposed to the disease [12].

While the genetic basis of PKD is understood, definitive clinical diagnosis occurs only after the evolution of established disease, when ultrasound is used to confirm the presence of renal cysts [13]. Therefore, we sought to translate the promising activity of etanercept in additional rodent genetic models where cysts are present at the onset of intervention.

## Methods

### Animals

A rat colony homozygous for mutation at the Pkhd1 locus (allele: PcK/Crljcrl-Pkhd1^pck^) was obtained from Charles River (Wilmington, MA). Male rats were employed in drug studies. Identification and characterization of this spontaneous mutant colony has been described [14]. A colony of PKD2 ws25/w183 compound heterozygous mice (referred to herein as ws25/-) kindly provided by Albert Einstein College of Medicine, and re-derived and maintained. Mice were genotyped by southern blots, and generally an equal number of male and female animals were apportioned to each study group. The Taconic Animal Care and Use Committee approved all study protocols. Animals had free access to water and standard diets.

### Experimental protocols

A summary of treatment and study design is included in Table 1. Rats were obtained at three weeks of age, and acclimated for one week preceding treatment. Etanercept (Enbrel, Amgen) was reconstituted regularly (vehicle 0.9% benzyl alcohol in sterile water) and single aliquots were used for a maximum of 4 days; dosing route was intraperitoneal (i.p.). Vehicle treatments were designed to match the excipient composition: 200 mM mannitol, 30 mM sucrose, 1 mM tromethamine in sterile water, pH7.4. Mozavaptan was synthesized (Focus Synthesis, San Diego, CA) and administered on a continuous basis via standard diet milled at the indicated concentrations on a per-weight basis. At the end of treatment, animals were anesthetized by carbon dioxide inhalation. Kidneys were removed and weighed; blood and serum was obtained by cardiac puncture method. The left kidneys were weighed and then fixed in 10% neutral-buffered formalin for 48Hrs, processing for histologic examinations; right kidneys were weighed and snap-frozen in dry ice for biomarker protein assay work.

**Table 1:** Treatment and methods summary for animal studies

**Study**	**Genotype**	**Age at treatment**	**Animals per arm**	**Treatments**	**Administration route**	**Dose**	**Schedule**	**Treatment duration**
ARPKD rat, short-term therapeutic intervention	PcK/Crljcrl-Pkhd1pck	4 weeks	10	Vehicle	NA	NA	NA	4 weeks
	PcK/Crljcrl-Pkhd1pck	4 weeks	10	Mozavaptan	i.p.	0.5, 5, 10 mg/kg	Once, every third day	4 weeks
ARPKD rat, Long-term therapeutic intervention	PcK/Crljcrl-Pkhd1pck	4 weeks	10	Vehicle	NA	NA	NA	8 weeks
	PcK/Crljcrl-Pkhd1pck	4 weeks	10	Mozavaptan	Milled chow	0.10%	ad libitum	8 weeks
	PcK/Crljcrl-Pkhd1pck	4 weeks	10	Etanercept	i.p.	5 mg/kg	Once, every third day	8 weeks
ADPKD (PDK2 ws25/-) mouse, therapeutic intervention	PKD2 ws25/-	12 weeks	14	Vehicle	NA	NA	NA	8 weeks
	PKD2 ws25/-	12 weeks	11	Mozavaptan	Milled chow	0.10%	ad libitum	8 weeks
	PKD2 ws25/-	12 weeks	15	Etanercept	i.p.	5 mg/kg	Once, every third day	8 weeks
ADPKD (PDK2 ws25/-) mouse, prophylactic intervention	PKD2 ws25/-	4 weeks	15	Vehicle	NA	NA	NA	8 weeks
	PKD2 ws25/-	4 weeks	15	Mozavaptan	Milled chow	0.05%	ad libitum	8 weeks
	PKD2 ws25/-	4 weeks	15	Etanercept	i.p.	5, 10 mg/kg	Once, every third day	8 weeks

Kidneys were embedded, sectioned and stained with hematoxylin and eosin (H&E) for histopathological evaluation of the degrees and severity of cyst formation. Tissues were sectioned longitudinally through the center keep the location of assessments constant. Cyst number was assessed by gross inspection and count from 3 random fields (×40 magnification) per animal, and averaged across cohorts.

Mice were weaned and allowed to acclimate to either 4 or 13 weeks of age before treatment began. Drug treatments and subsequent tissue, blood and any biomarker analyses were performed as described above.

In rats, water intake was assessed by periodically weighing cage bottles, and urine output was measured following forced manual voiding. In mice, urine production was assessed using metabolic cage monitoring; recording sessions were acquired in two separate morning and evening sessions, for two hours each. Mouse water consumption was monitored through periodic cage bottle weighing.

### Kinase signaling analysis

Kinase signaling was assessed on homogenized kidney lysates using a multiplex, bead-based suspension protein array service (AssayGate Inc, Ijamsville, MD).

### Serum chemistry

Serum urea nitrogen levels and creatinine were measured using a Beckman autoanalyzer and standard colorimetric assessments (Beckman Instruments, Fullerton, CA).

### Statistical analyses

Multiple group comparisons were performed using a one-way ANOVA with post-test according to Dunnet. P < 0.05 was considered statistically significant. Values are expressed as means ± SEM.

## Results and discussion

**Figure 1:**
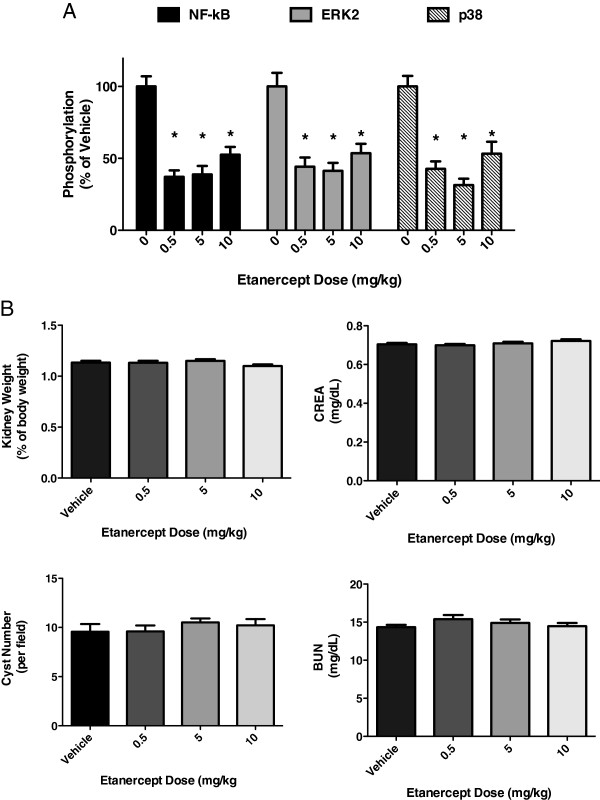
**Short**-**term (4 weeks) entanercept pharmacodynamics and efficacy in pck rats. (A)** Stress kinase phosphorylation from kidney tissue lysates (N = 4) is significantly reduced following 4 weeks dosing with etanercept at various levels. **(B)** Kidney weight, cyst number, serum urea nitrogen and creatinine are unchanged following etanercept treatment for 4 weeks (N = 10).

Initially, we studied the pck rat disease model, which harbors homozygous null mutations in the orthologous PKHD1 susceptibility locus: this model of autosomal recessive PKD (ARPKD) was previously used to study the potential therapeutic effects of vasopressin and mTOR/AKT pathway inhibitors [4,15]. We first established an optimal dosing protocol for etanercept, as the drug could display unexpected pharmacokinetic and pharmacodynamic effects in certain rodent models. These pilot studies showed that, in kidneys analyzed following 4 weeks of treatment, as little as 0.5 mg/kg etanercept dosed every three days was sufficient to markedly reduce both NF-kB (p65-S536) and p38 stress kinase pathway signaling, which are established mechanistic biomarkers of TNF-a signaling [16] (Figure 1A).

Having proven that etanercept could reduce relevant inflammatory signaling in the kidneys of pck rats, we next studied whether the drug could improve established markers of renal function and PKD pathology. In 4-week old animals treated for one month, gross kidney weights, as well as average cyst number quantified after histological examination, were unchanged by etanercept doses as high as 10 mg/kg (Figure 1B). Similarly, the renal function markers blood urea nitrogen (BUN) and creatinine remained unchanged following treatment (Figure 1B). Other investigators have noted that rodent models of PKD are sensitive to the timing and duration of treatment [6]. Therefore, we tested prolonged etanercept dosing at 5 mg/kg every 3 days for 8 weeks; in this background, we additionally tested continuous administration of the vasopressin antagonist mozavaptan at pharmacologically relevant doses. We noted that mozavaptan induced aquaretic effects, which confirmed that our studies could properly recapitulate known pharmacologic effects of this drug class. Still, in the more chronic treatment setting, neither etanercept nor mozavaptan proved efficacious against multiple therapeutic markers of PKD. Surprisingly, both gross kidney weight and cyst number were moderately increased in both drug treatment arms (Table 2). While BUN was largely unchanged following treatment, animals treated with either etanercept or mozavaptan had blood creatinine levels greater than untreated animals (Table 2). Given the previously observed effects of vasopressin antagonists in preserving kidney volume and reducing cysts [4,6], though, we conclude etanercept is largely ineffective in this model of ARPKD.

**Table 2:** **Efficacy of long**-**term** (**8 week**) **treatment with mozavaptan or etanercept in pck rats**^**a**^

**Treatment**	**Kidney weight (% of bw)**	**Cyst number (per field)**	**BUN (mg/dL)**	**CREA (mg/dL)**	**Water consumption**^**d **^**(mL/rat/day0029)**
Vehicle	1.05 ± 0.023	9.2 ± 0.71	14.9 ± 0.51	0.63 ± 0.05	42.96 ± 1.96
Mozavaptan (0.1%)	1.12 ± 0.04	12.1 ± 1.4	16.2 ± 0.39	0.71 ± 0.03^b^	63.88 ± 0.56^c^
Etanercept (5 mg/kg)	1.16 ± 0.036	12 ± 1.3	16.4 ± 0.4	0.71 ± 0.06^b^	38.95 ± 1.40

^a^Data shown are group average ± *SEM*, N=10.

^b^Comparison between vehicle and treatment group(s), p < 0.05.

^c^Comparison between vehicle and treatment group(s), p < 0.001.

^d^Water consumption measured over final week of treatment.

Still, it remained possible etanercept could be effective in models of autosomal dominant PKD (ADPKD). Here, we studied mice hemizygous for the PKD2 ws25 allele; this latent disease model rapidly develops cysts in a stochastic fashion, following somatic recombination and loss-of-heterozygosity [17]. We first tested etanercept in 13 week-old PKD2 ws25/- animals, which have previously been shown to harbor overt signs consistent with polycystic kidney disease. Following dosing for 8 weeks, 5 mg/kg etanercept did not improve kidney weight, cyst number, or BUN. As in pck rats, we also administered 0.1% mozavaptan continuously via diet; perhaps surprisingly, this treatment also proved ineffective across the multiple parameters investigated (Table 3).

**Table 3:** **Prophylactic and therapeutic administration of mozavaptan or etanercept in PKD2**(^**ws25 **/-)^**mice**^**a**^

**Treatment**	**Age at treatment**	**N**	**Kidney weight (% of bw)**	**Cyst number (per field)**	**BUN (mg/dL)**	**Urine production**^**b **^**(mL/mouse)**	**Water consumption**^**b **^**(mL/mouse/day)**
Vehicle	13 weeks	14	1.85 ± 0.17	6.7 ± 0.7	20.6 ± 1.2	0.99 ± 0.20	4.0 ± 0.04
Mozavaptan (0.1%)	13 weeks	11	1.74 ± 0.11	6.9 ± 1.1	23.6 ± 2.0	4.93 ± 1.64^c^	11.1 ± 0.35^c^
Etanercept (5 mg/kg)	13 weeks	15	1.75 ± 0.09	6.5 ± 0.8	20.1 ± 1.5	NA	4.0 ± 0.06
Vehicle	4 weeks	15	1.96 ± 0.15	7.0 ± 0.7	24.8 ± 2.7	NA	6.1 ± 0.18
Mozavaptan (0.05%)	4 weeks	15	1.78 ± 0.07	7.7 ± 0.8	26.1 ± 2.1	NA	9.6 ± 0.35^c^
Etanercept (10 mg/kg)	4 weeks	15	1.84 ± 0.2	5.5 ± 0.6	21.9 ± 1.6	NA	5.9 ± 0.16
Etanercept (5 mg/kg)	4 weeks	15	1.77 ± 0.17	6.2 ± 0.7	23.2 ± 2.0	NA	5.8 ± 0.18

^a^Data shown are group average ± *SEM*.

^b^Urine production over 4 hours monitoring in metabolic cage; separate measure of daily water consumption, by bottle weighing.

^c^Comparison between vehicle and treatment group, p<0.05.

Finally, we studied etanercept initiated at the earliest time point achievable in *in*-*vivo* settings. Starting at 4 weeks of age, we administered either 5 or 10 mg/kg etanercept to PDK2 ws25/- mice once every three days for two months. As in pck rats, etanercept proved ineffective in treating various aspects of PKD: kidney weight, and cyst number were not reduced following treatment, and BUN also remained unchanged (Table 3). In total, we conclude that, in settings that would reveal either therapeutic or disease-modifying activities, etanercept is largely ineffective at treating the key pathologic and physiologic dysfunctions of PKD.

## Conclusions

Considering our data in the context of prior mechanistic and clinical studies, we suggest several key conclusions. First, and most importantly, etanercept, and likely TNF-α blockade in general, appears to be a relatively poor candidate therapeutic approach for study in future clinical trials. Clinical trials of vasopressin and mTOR antagonists have shown marginal efficacy and important side effects, despite showing promising preclinical efficacy for several of our investigated parameters, in rodent models closely related those employed here. Our studies were powered to discern therapeutic effects similar in magnitude as those drugs currently in clinical testing. Therefore, TNF-α blockade seems less attractive compared to other candidate therapeutic mechanisms both currently and prospectively being assessed for clinical translation.

Second, our study underscores how fundamental molecular and cellular events observed in preclinical research can be difficult to translate into treatments for disease biology as it presents during common clinical practice. Current clinical criteria dictate that, even in the presence of strongly predictive genetic risk, formal diagnosis of ADPKD requires the observation of cysts via ultrasound exam [13]. By contrast, Li et al. showed that, in young mice heterozygous for PKD2 loss, cyst formation could be prevented by prophylactic etanercept [12]. While promising, the analogous application of this observation into clinical practice would imply life-long dosing and consequent immunosuppression, initiated in very young patients with a confirmed genetic background of PKD. Preventative therapy along these lines would likely be difficult to test in clinical trials, and could also prove challenging in everyday clinical practice.

Lastly, our data suggest additional, relevant hypotheses concerning disease pathology and treatment in PKD. Patients presenting with PKD are likely phenotypically null for essential polycystin gene function(s)--single PKHD1 or “two-hit” PKD2 mutations both damage renal cell function sufficiently to cause disease. While Li et al. suggested that TNF-α blockade could rescue residual PKD2 activity, our work suggests that later pathophysiological events in PKD are largely TNF-α independent. Also, as TNF-α has been long appreciated as a key, nodal point of inflammatory signaling, it seems possible that other anti-inflammatory approaches may also prove ineffective in PKD—specifically, approaches that simply decrease NF-kB or p38 stress kinase signaling (as in our studies) may not sufficiently modify the underlying pathophysiology of PKD.

In summary, our studies have explored the possible therapeutic benefit of TNF-α blockade in rodent models of PKD previously used to nominate agents for interventional clinical trials. We suggest our data de-prioritize this putative disease mechanism for future clinical testing in settings of established disease. It remains possible, however, that alternate anti-inflammatory approaches could be safe and effective treatments for PKD, and that chronic TNF-α inhibition could prevent the emergence of PKD in patients who are genetically “at-risk”, but harbor occult disease.

## Competing interest

The authors declare that they have no competing interest.

## Authors’ contributions

SS designed the experimental approach and studies, and coordinated experimental execution. JR analyzed data. JR and SS wrote the manuscript. Both authors read and approved the final manuscript.

## Pre-publication history

The pre-publication history for this paper can be accessed here:

http://www.biomedcentral.com/1471-2369/14/233/prepub
